# Parent and Primary Care Clinician Perceptions About Pediatric Hypertension

**DOI:** 10.1001/jamanetworkopen.2024.51103

**Published:** 2024-12-13

**Authors:** Abbas H. Zaidi, Erica Sood, Sarah De Ferranti, Samuel Gidding, Varsha Zadokar, Jonathan Miller, Anne Kazak

**Affiliations:** 1 Nemours Children’s Health Cardiac Center, Wilmington, Delaware; 2 Department of Pediatrics, Sidney Kimmel Medical College, Thomas Jefferson University, Philadelphia, Pennsylvania; 3 Division of Behavioral Health, Nemours Children’s Health, Wilmington, Delaware; 4 Nemours Children’s Health Center for Healthcare Delivery Science, Wilmington, Delaware; 5 Division of Ambulatory Cardiology, Department of Cardiology, Boston Children’s Hospital, Boston, Massachusetts; 6 Department of Pediatrics, Harvard Medical School, Boston, Massachusetts; 7 Department of Genomic Health, Geisinger Medical Center, Danville, Pennsylvania; 8 Department of Pediatrics, Nemours Children’s Health, Wilmington, Delaware

## Abstract

**Importance:**

Despite published guidelines and a prevalence of pediatric hypertension of approximately 3%, less than 25% of affected children are identified, with 60% not receiving any intervention despite detection. Understanding the knowledge and perceptions of parents or caregivers and health care teams is crucial to identify factors that may contribute to these unacceptably low detection rates.

**Objective:**

To examine the knowledge and perceptions of parents and health care teams regarding pediatric hypertension to identify common themes contributing to low hypertension detection.

**Design, Setting, and Participants:**

This qualitative study, interviewed parents of children diagnosed with hypertension who did not follow up within 1 year after diagnosis and health care clinicians (medical assistants, nurses, managers, pediatricians, and nurse practitioners) across 10 clinics in Delaware and Pennsylvania between November 1, 2022, and March 31, 2023.

**Main Outcomes and Measures:**

With the use of an inductive thematic approach, codes were developed and themes identified by multidisciplinary research investigators.

**Results:**

A total of 38 stakeholders (mean [range] age, 43 [25-64] years; 33 [86%] female), including 13 parents and 25 health care clinicians, were interviewed. The parent sample was diverse based on race (5 [46%] Black, 5 [38%] White, and 3 [23%] other race), ethnicity (5 [38%] Hispanic and 8 [62%] non-Hispanic), and Child Opportunity Index (5 [38%] very low or low, 3 [23%] moderate, and 5 [38%] high or very high). Parents and clinicians demonstrated awareness of the significance of pediatric hypertension. Parents thought blood pressure checks were important; however, clinicians often had competing priorities during annual visits. Both groups expressed skepticism about high blood pressure readings, attributing them to situational factors or white coat syndrome. Parents and clinicians shared concerns about medication use and preferred lifestyle change. Parents advocated for further testing, whereas clinicians exhibited varying perspectives on additional diagnostics, emphasizing targeted testing strategies and cautious medication approaches.

**Conclusions and Relevance:**

In this qualitative study of parents and clinicians, both groups were skeptical of blood pressure readings and expressed concerns about medication use, with a preference for lifestyle changes over medication. Improving detection may require better measurement tools, reassurance about medications, and clear communication on the role of nonpharmacologic treatments, while future interventions should incorporate the perspectives of both parents and clinicians to develop practical strategies for managing pediatric hypertension.

## Key Points

**Question:**

Are perceptions of parents and health care teams associated with low detection and management of pediatric hypertension?

**Findings:**

This qualitative study of 38 parents and clinicians in a multisite pediatric health care system found that both groups recognized the importance of pediatric hypertension but were skeptical of blood pressure readings and concerned about medication use. Although parents prioritized annual blood pressure checks, clinicians often downplayed high blood pressure in clinic, with both groups favoring lifestyle changes over medication, highlighting the need for improved communication around nonpharmacologic treatments.

**Meaning:**

These findings suggest that skepticism about blood pressure readings, medication concerns, and preference for nonpharmacologic interventions among parents and clinicians contribute to low pediatric hypertension detection and management, necessitating incorporation of their perspectives for improvement.

## Teaser

This qualitative study examines parents’ and primary care clinicians’ perceptions of pediatric hypertension across diverse practice settings and the association of these perceptions with detection and management of pediatric hypertension.

## Introduction

Pediatric hypertension, with a prevalence of approximately 3%,^1,2,3,4^ poses significant risk for conditions such as kidney failure and cardiovascular diseases in adulthood.^5,6,7^ American Academy of Pediatrics guidelines recommend screening for hypertension beginning at the age of 3 years^1^; however, less than 25% of affected children are identified during pediatric visits,^1,8,9^ with up to 60% receiving no intervention even after hypertension detection,^10^ demonstrating significant gaps in implementation. Investigation into parents’ and health care clinicians’ perspectives can inform and help close guideline implementation gaps by identifying the perceptions that may contribute to these unacceptably low detection rates.

Notably, a significant gap exists in the current understanding of clinician and parent perspectives, especially of those from diverse backgrounds, encompassing not only race and ethnicity but also diversity in socioeconomic status, language, and the roles and experience levels of clinicians.^9,11^ The lack of inclusion of these perspectives misses valuable opportunities for improving pediatric hypertension detection. This qualitative study aimed to fill this gap by examining the knowledge and perceptions regarding pediatric hypertension in parents and primary care clinicians across diverse practice settings, with attention to social determinants of health.

## Methods

For this qualitative study, we recruited participants from 10 primary care clinics across Nemours Children’s Health (NCH) in Delaware and Pennsylvania between November 1, 2022, and March 31, 2023. The 10 clinics were identified through a 2-step process. First, we identified clinics with significant gaps in follow-up care for patients diagnosed with hypertension, determined by the percentage of patients who did not receive follow-up care within a year (range, 0.9%-28%) (eTable 1 in Supplement 1). Second, we calculated each clinic’s Childhood Opportunity Index (COI) 2.0 to evaluate the socioeconomic status of the regions served. The COI quantifies 29 indicators of neighborhood conditions, with scores from 1 to 100, with higher scores indicating more favorable neighborhood opportunities.^12,13^ To facilitate our analysis, clinic sites were categorized into 3 COI groups: very low or low (COI, <40), moderate (COI, 40-<60), and high or very high (COI, ≥60). All parents provided written informed consent. This study was approved by the NCH Institutional Review Board. Qualitative methods were guided by and reported in compliance with the Consolidated Criteria for Reporting Qualitative Research (COREQ) reporting guideline.^14^

### Participants

#### Parents

Parents of children with gaps in follow-up care for a hypertension diagnosis at primary care clinics were included. To ensure a diverse sample, parents were recruited based on race, ethnicity, language preference (English or Spanish), and COI.^12^ Eligible parents were primary caregivers of patients aged 6 to 17 years who visited clinics between January 1 and December 31, 2021, with a diagnosis of hypertension or related *International Statistical Classification of Diseases and Related Health Problems, Tenth Revision* (*ICD-10*) codes (eg, elevated blood pressure [BP], stage 1 hypertension, or stage 2 hypertension), with stage 2 BP recorded at 2 or more previous visits, and with no follow-up within 1 year from the last documented stage 2 BP. We focused on patients with diagnostic codes related to hypertension to exclude cases of inaccurately measured BP or situations in which clinicians did not identify the BP as high. Exclusions included patients with developmental delay, autism, or medications affecting BP, which could result in falsely elevated readings, or those receiving antihypertensive therapy.^15,16^

#### Health Care Clinicians

Health care clinicians from the 10 pediatric primary care clinics were systematically recruited, including physicians, medical assistants, nurses, and nurse managers responsible for clinic operations (eTable 1 in Supplement 1). Recruitment was facilitated through email invitations, and clinicians were selected to ensure a diverse representation of roles across sites with varying COIs and follow-up care gaps. Clinicians from each site reflected key clinical roles, aiming to capture a wide range of perspectives. We did not collect data on race and ethnicity because these data can sometimes be perceived as sensitive information, particularly if clinicians feel that disclosing this information could influence how their input is interpreted or be traced back to them.

### Interview Methods

Parents and health care team members participated in 30- to 45-minute telephone interviews conducted by a trained research coordinator (V.Z.) and/or the principal investigator (A.H.Z.). Interview guides were based on Consolidated Framework for Implementation Research (CFIR),^17^ a theoretical framework that evaluates factors influencing successful implementation of health practices. The CFIR is organized into 5 major domains: innovation (characteristics of the practice change), outer setting (external influences), inner setting (health institutional context), characteristics of the individual (people involved), and implementation process (steps taken to adopt the new practice change). Each domain contains multiple constructs representing specific elements or concepts that facilitate a deeper exploration of the factors influencing implementation (eg, constructs within the innovation domain include innovation source, which refers to whether the innovation was developed internally or externally, which can affect how easily it is adopted). Interview questions for parents and clinicians were mapped onto the 5 CFIR domains and the respective constructs that applied to this study (eTable 2 in Supplement 1).

Parents were contacted by telephone and clinicians via email. Parents who were not present for or did not recall their child’s last visit for hypertension were excluded. Eligible parents signed electronic consent forms and scheduled a convenient interview time. A Spanish interpreter was engaged throughout the study to ensure effective communication with Spanish-speaking families. Spanish interpreters were present during the interviews to assist with real-time translation. Parents completed a demographic survey with questions on race, ethnicity, highest educational level, household income, and health insurance via Research Electronic Data Capture (REDCap).^18^ The patients’ clinical characteristics were extracted from the electronic medical record and made available during the interview to help the parent recollect aspects of the visit. Clinicians were asked basic questions about their educational background, role, tenure, and general clinic workflow. Interviews were recorded and transcribed, and deidentified transcripts were uploaded into Dedoose, version 9.0.106 (SocioCultural Research Consultants LLC). Parents received a $50 gift card for participation, whereas the clinicians did not receive compensation.

### Statistical Analysis

Qualitative data were analyzed using an inductive thematic approach focusing on participants’ perceptions and feelings. A pediatric cardiologist (A.H.Z.) and psychologist (E.S.) conducted open coding, developing codebooks iteratively through transcript reviews and discussions. Three consecutive parent interview transcripts and 4 consecutive clinician interview transcripts were coded independently by the 2 coders (A.H.Z. and E.S.) with intercoder reliability (pooled Cohen κ coefficients) of 0.87 and 0.91, respectively. The remaining transcripts were divided between the 2 coders. Excerpts coded as knowledge and perceptions were organized into key themes, supported by direct quotations. Collaborative analyses through debriefing sessions were performed to validate interpretations. Themes were subsequently aligned with the CFIR framework.

## Results

### Sample Characteristics

A total of 38 stakeholders (mean [range] age, 43 [25-64] years; 33 [86%] female), including 13 parents and 25 health care clinicians, were interviewed between November 1, 2022, and March 31, 2023. The parent sample was diverse in race (5 [46%] Black, 5 [38%] White, and 3 [23%] other race, including 1 who identified as Puerto Rican, 1 who identified as mixed race [Black and American Indian], and 1 who identified as Black and other race), ethnicity (5 [38%] Hispanic and 8 [62%] non-Hispanic), and Child Opportunity Index (5 [38%] very low or low, 3 [23%] moderate, and 5 [38%] high or very high) (Table 1). The health care team sample was diverse with respect to professions (6 physicians in charge or regional chief [24%], 5 primary care physicians [20%], 6 nurse managers [24%], 3 medical assistants [12%], 4 nurses [16%], and 1 advanced practice nurse [4%]) (Table 1).

**Table 1.** Sociodemographic Characteristics of Participants

Characteristic	No. (%)
**Parents (n = 13)**	
Sex	
Female	11 (85)
Male	2 (15)
Race	
Black	5 (38)
White	5 (38)
Other^a^	3 (23)
Ethnicity	
Hispanic	5 (38)
Non-Hispanic	8 (62)
Language	
English	9 (69)
Spanish	4 (31)
Highest educational level	
Less than high school diploma	2 (15)
High school graduate or equivalent	4 (31)
Some college	2 (15)
College degree or higher	5 (38)
Household income, $	
<25 000	2 (15)
25 000-49 000	3 (23)
50 000-99 000	3 (23)
100 000-149 000	2 (15)
≥150 000	2 (15)
No answer	1 (8)
Health insurance	
Public	6 (46)
Private	6 (46)
Other	1 (8)
Child Opportunity Index	
Very low or low	5 (38)
Moderate	3 (23)
High or very high	5 (38)
**Health care clinicians (n = 25)**	
Age group, y	
25-34	4 (16)
35-44	9 (36)
45-54	5 (20)
55-64	7 (28)
Sex	
Female	22 (88)
Male	3 (12)
Role	
Physician in charge or regional chief	6 (24)
Primary care physician	5 (20)
Nurse manager	6 (24)
Medical assistant	3 (12)
Nurse	4 (16)
Advanced practice nurse	1 (4)
Educational background	
Medical degree (MD, DO)	11 (44)
Bachelor’s degree (nursing, psychology, behavioral science)	7 (28)
Master’s degree (nursing, psychology)	4 (16)
Associate’s degree	3 (12)
Length of service at NCH, y	
<5	5 (20)
5-10	10 (40)
11-15	3 (12)
16-20	3 (12)
>20	4 (16)
Clinic location’s Child Opportunity Index	
Very low or low	15 (60)
Moderate	4 (16)
High or very high	6 (24)

Abbreviation: NCH, Nemours Children’s Health.

^a^ Other race is representative of 1 parent who identified as Puerto Rican, 1 parent who identified as mixed race (Black and American Indian), and 1 parent who identified as Black and other race.

Five overarching themes related to pediatric hypertension were identified, highlighting parallel concerns of both parents and clinicians across various CFIR domains. These themes, subthemes, and representative quotations are given in Table 2. Thematic saturation was achieved, with each theme supported by more than two-thirds of parents and clinicians. The major themes and their contributory CFIR domains are given in eTable 3 in Supplement 1. The influences from these perceptions on parent behavior is shown in the eFigure in Supplement 1.

**Table 2.** Parent and Health Care Clinician Themes and Representative Quotations Related to Pediatric Hypertension

Subthemes	Parent or clinician quotations
**Major theme 1: knowledgeable on pediatric hypertension (92% parents, 88% clinicians)**	
Parent knowledge subthemes (eg, can have symptoms such as headaches or fainting; asymptomatic or silent killer; dangerous, hospitalization, causes death; harms organs, including heart and brain)	I know that it’s like a silent condition like a silent illness, that can kill you all of a sudden. You can really have a spike… and it can cost you your life. –Parent 14
	My father, patient’s father, patient’s grandmother… a lot of people in my family that have high BP on both my side and patient’s father’s side. So how do I feel about it? I mean, from the medical standpoint, I understand… it can cause harm to organs. –Parent 7
	I have high BP. I was put on BP medicine at 32. My mother had it. My mother is deceased…. She was on medication for it and my grandmother was on medication for it as well. –Parent 10
Clinician knowledge subthemes (eg, end-organ damage; silent killer; linking hypertension with obesity and pandemic)	I think it’s very important. I would say very important. I would imagine, you know, if even if a 12-year-old has hypertension. It does put strain on their end organs. So yeah, I think it’s just as important that as it is on the adults. –Clinician 9
	It affects all multiorgan system for their future…. It’s important to prevent the damage micro, damage to the organs for future. –Clinician 22
	I think it’s very important. I think that word has some weight and impact. If you tell a family, “The BP is high today, I’m worried about this, this is something we need to pay attention to.” Because you know the silent killer, right, they don’t know they have high BP. I think parents are aware of, like high BP being a problem some more than others. And then when you tell them, ’Your kids BP is high,’ it has some impact. –Clinician 10
	We are seeing more of it in our clinic just from what I’ve heard from my providers. You know, over the last 3 years, kids are facing more health challenges following the pandemic and I think it’s definitely something along with obesity that we need to be addressing. –Clinician 12
	I do feel like obesity is playing more of a factor in the adolescents which seems to be also play a factor on the BP. –Clinician 24
	We have a lot of obese kids, we have kids that have poor diets, poor home lives. They have lots of insecurities in terms of like food, shelter, money you know things like that and so all these things taken into consideration we have and, but we do have a lot of kids that are obese and so it’s a problem. –Clinician 1
	I think it’s now becoming a problem, especially for older kids. I have noticed that kids that are either in middle school or high school especially if there’s concomitant weight issues like overweight, obesity and morbid obesity. I am seeing now since I started practice that I’m seeing a lot more kids with hypertension. –Clinician 16
**Major theme 2: prioritization of pediatric BP (100% parents, 72% clinicians)**	
Parent prioritization subthemes (eg, importance for health maintenance; to prevent future issues, such as organ damage; concern due to young age and family history)	Because it does run in our family and it is a concern. I mean, he’s 16 years old. So they have to worry about things like that. My nephew is 23 and he has to take medication for his BP. –Parent 4
	I think it [BP checks] should be like once-a-year physicals… at least once a year like a flu shot…. It’s better to get it detected early so you can say, “Look you need to make these changes.” Also, you know, tell them [patient/families] what’s going on so they can know if you don’t make these changes and this could happen or you know, whatever the situation calls for. –Parent 2
Clinician lack of a priority subthemes (eg, asymptomatic; focus on other health conditions; not part of routine workflow)	I don’t think it’s [high BP] one of the most important things we focus so much on. They have to have a physical, immunizations…. So, it’s not on top of the list for sure… –Clinician 9
	This is silent killer. This is not the patient with asthma with pulse O2 of 88, very tight, gasping for air to the emergency room… I don’t have any patient in all my years with a hypertensive crisis. Bottom line is it’s a silent killer… I am unfortunately pushing this to my colleagues in family medicine and internal medicine… even though you have readings here of 140/95 from time to time… the patient has no symptoms (… no blurry vision… neck pain… doesn’t feel dizzy… doesn’t have epistaxis). –Clinician 15
	Hypertension is probably less important because there’s so many other factors as far as treating depression especially in our adolescent visits, that is taking up a lot of time in the visit… making sure that routine preventative care, vaccines, STD testing is being obtained and things like that…. Even in our smart sets and things that we use, BP monitoring and counseling is not really a part of our system flow, whereas talking about sex risks and drug use and mental health is all built into the workflow of our smart sets and our notes. –Clinician 20
**Major theme 3: lack of trust of high BP measured in clinic (77% parents, 100% clinicians)**	
Parent lack of trust subthemes (eg, young age; lack of PCP concern, trust in care team; nervous or situational)	And I’m like, dude, you will be fine. You’re 16 years old, your body bounce back. It’s not a problem. It’s not like you’re 40. You know, it’s not. –Parent 2
	The information [on high BP noted] was not verbalized. So, I did not even look at the paper because I mean he’s 15 at this point he’s had at least 20 physicals, you know, from infancy to now. If they’re not telling me that anything’s important, I’m not going to go looking for anything… if they gloss over it like “Ohh, they’re just probably nervous, this or that, and the other.” I’m not gonna look at that as anything significant or serious. –Parent 7
	We did not have a discussion in reference to it being high like or consecutively being high, but she did say it was a little elevated when he took his BP due to maybe being nervous. –Parent 10
Clinician hesitancy subthemes (eg, white coat syndrome; attributing high BP to other factors; not believing high BP in children)	The kids are stressed like we’re stressed and sometimes just come into the doctor’s office stressful and that could raise the BP. –Clinician 7
	It is a little bit hard to tease out sometimes because most kids are nervous when they come to our office, particularly because of vaccines and things like that. So sometimes it’s really hard for us to make a judgment on true hypertension vs white coat syndrome. –Clinician 3
	I think there is a lot of white coat hypertension in [pediatrics], and you don’t want to just dismiss it as that, but we probably have a tendency to do that a lot. –Clinician 10
	I know it [BP] is very important, but often I think it’s overlooked… it’s not always addressed properly that’s my concern. It’s always blamed on white coat. And I think people don’t think of kids as having high BP so it’s always blamed on something else. –Clinician 2
**Major theme 4: concerns about medication use (85% parents, 76% clinicians)**	
Parent medication subthemes (eg, want to lower it naturally; adverse effects; child is young)	Because I would definitely like to know the side effects to make sure it doesn’t harm him in any way and also later on down the line with him getting, it’s more so that this is being in his system and also like I said being so young, I want to make sure it’s something that’s needed, not just no placebo or anything. –Parent 4
	I was more concerned about how his BP was going to be lowered. I didn’t want him to take any medications. I just wanted it to be a natural way of doing it…. You become medication dependent or you would have to use medication for the rest of your life. And that’s something that we don’t want to happen for him. –Parent 13
	Well. Yes, if he doesn’t reduce it [BP] then it can have a negative effect on his health and we don’t want him to be on medications that he has to depend on the rest of his life just to regulate his BP. So, we’re trying to avoid that… we don’t want that to happen. –Parent 9
	Medications have side effects and then this one has a side effect. It might not be a good side effect. You got to try something else and you try this and try that. I’ve been down that road. I have allergies too and I found I’m allergic to certain medications. I’ll try this, this happened, or you have to be on it for the rest of your life and some medications. If you’re on it for a long time, getting off of it is not a great experience either, like you have to wean off of it or you can’t come off of it because whatever you’re on and like your body is so used to it being on there. That’s all. –Parent 9
Clinician medication subthemes (eg, preference to avoid medications; adverse effects; lack of patient acceptance; lack of patient adherence)	That is normally the first question they [parent or family] always ask. “Do they need to take a medication? Because I had a medication and it made me cough a lot. And I don’t want my kid to be on that medication.” –Clinician 20
	It is the last resort. It should be the last resort—medications in chronic use. They had side effects… We should work with the family to control the BP when it’s possible without any medications… because again few patients I have with medications prescribed by a nephrology and cardiology, they do not take the medication. –Clinician 15
	I don’t typically prescribe them myself. I guess I’ve personally never I think recently been in a situation where I felt like essential hypertension needed medical treatment. And if it’s secondary to something else, like, for example, renal disease, then I leave that up to the nephrologist to decide. But I’ve so far never really been in a situation where I felt like they needed antihypertensive that I was going to prescribe without the support of a specialist. –Clinician 17
	Because we don’t want to put kids on medication that shouldn’t be on medication. –Clinician 8
	I do not believe that the key is medication. Because the patients prove me that they will not take the medication and a study showed that 66% of adult Americans with hypertension, they do not take the medication properly or they do not take the medication. Period. –Clinician 15
**Major theme 5: additional testing for hypertension (100% parents, 92% clinicians)**	
Parent additional testing subthemes (eg, advocating for additional testing)	I’m 100% on board with that. If any of those things had been offered or even suggested, he would have had them. –Parent 8
	I think it would be important to take all those steps (including testing) to determine or first of all to confirm that he truly has high BP, impacting his health. –Parent 5
	I will feel more comfortable and more assured with the tests and making sure that everything is OK with him as well while he is being tested. –Parent 14
Clinician additional testing subthemes (eg, belief that further testing is not useful; variability in further testing; selective testing; referral preferred)	I guess sometimes I do labs—it’s just so infrequent. I guess I could do labs, but most of the time we just refer. –Clinician 14
	I don’t think there’s much value there [in doing further testing]. I mean there is like some for echocardiogram. I’m not sure for like for blood work and all but yes, they are to rule out the secondary causes first. Because primary hypertension is not the most common one in pediatrics. –Clinician 22
	Well, also it depends on the patient and I’m working with many of the patients with elevated readings. They have obesity or morbid obesity. We know that for these patients, most of them, they work up for high BP and it comes back normal and the solution for the problem is to lose your weight and exercise but usually doesn’t happen. And unfortunately, we have some of these patients that, I have 2 on the top of my head now. Two teenagers, 1 boy and 1 girl, who have severe obesity, and both of them were started on BP medication and none of them are taking the medication. OK, I think they are important. OK, but I think we are going to overdo it if we don’t have accurate readings of the BP. –Clinician 15
	I would say no, and I refer to specialty other than I would say outside of obesity. So, for those patients, I’m really doing an obesity workup. Not a blood hypertension workup. –Clinician 3

Abbreviations: BP, blood pressure; PCP, primary care physician; STD, sexually transmitted disease.

### Knowledgeable About Hypertension (CFIR Innovation Domain)

Many parents and clinicians demonstrated awareness of the significance of addressing pediatric hypertension, aligning closely with the constructs within the innovation domain of the CFIR. They recognized the potential effect on vital organs, such as the heart, kidneys, and brain. Clinicians linked the increase in pediatric hypertension to broader health challenges, including increased rates of obesity after the COVID-19 pandemic and the relative advantage of early intervention in mitigating long-term health implications. Parents and clinicians recognized the complexity of hypertension diagnosis, particularly its often asymptomatic presentation and the association between hypertension and significant morbidity later in life. Parents expressed their understanding of the risks associated with high BP as well as management and treatment, drawing connections to familial histories of hypertension. Parents mentioned gaining this knowledge through their own personal experiences and not from the clinicians during clinic visits. In fact, parents did not feel it necessary to ask any questions from the clinicians given their confidence in their hypertension knowledge.

### Prioritization of Pediatric Hypertension (CFIR Inner Setting Domain)

Although both parents and clinicians shared the perception of pediatric hypertension as important, there were differences in how it was prioritized. Many parents expressed worry and concern due to a family history of hypertension, considering hypertension a significant issue for their children. Parents emphasized the importance of checking BP during annual physical examinations, analogous to an influenza shot, emphasizing the value of early detection and lifestyle modifications. However, clinicians often downplayed the significance of high BP, stating it is not a focus during clinic visits, especially compared with other aspects of the physical examination and immunizations. Clinicians emphasized that one reason for this lack of prioritization is the silent nature of hypertension, making this disease process less urgent for clinicians overall compared with conditions with more overt symptoms. In addition, clinicians indicated that BP monitoring and counseling are not components embedded within their workflows, making hypertension less prioritized compared with immunizations for pediatric patients or mental health and substance abuse problems for adolescent patients.

### Lack of Trust in High BP Noted in Clinic (CFIR Outer Setting Domain)

Despite recognizing hypertension as a significant concern, parents and clinicians both reported that high BP measured during routine visits was likely erroneous. Parents commonly attributed elevated readings in the clinic to their child’s young age or issues in obtaining an accurate BP and believed the body would naturally resolve it over time. They also attributed elevated readings to situational factors, such as being nervous, and downplayed the importance of high BP in children. The perception that it was situational was shared by the clinicians, who indicated that the high BP was likely to be falsely elevated due to the patient being anxious. Furthermore, parents reported trusting their physicians and health care team and felt confident in the information provided during visits. Therefore, trust in high BP values was compromised if the information was inadequately communicated or if the presence of high BP was not emphasized during routine checkups. This skepticism was particularly notable among parents who had established long-term relationships with their clinicians, reflecting the influence of local conditions on parental perceptions. Many clinicians acknowledged the challenge of distinguishing true hypertension from a child being nervous or white coat syndrome. Clinicians also noted that although high BP was necessary to assess, it was often overlooked, attributed to inaccuracies in obtaining the BP or the equipment used.

### Medication Use in Pediatric Hypertension (CFIR Characteristics of the Individual Domain)

Although a few parents and clinicians were open to the idea of using antihypertensive medications, many parents and health care clinicians expressed reservations about medication use in pediatric hypertension. Parents and clinicians prioritized nonpharmacologic approaches, such as dietary changes, increased physical activity, and weight management. Parents and clinicians emphasized a strong preference to avoid medications, citing concerns about adverse effects. This reluctance by clinicians was reinforced by a perception that parents would resist medication adherence due to concerns about adverse effects and the stigma associated with long-term medication use in children. Clinicians preferred to defer to specialists for medication initiation, feeling uncomfortable prescribing antihypertensives to their patients.

### Further Testing in Pediatric Hypertension (CFIR Implementation Process Domain)

All parents expressed interest in diagnostic testing for pediatric hypertension and deemed it essential for an accurate diagnosis. In contrast, clinicians had varying practices when it came to additional diagnostic testing, but most emphasized limited value in testing and concerns about potential overtesting. Most clinicians preferred to refer patients to subspecialties for testing and diagnosis instead of initiating or completing a diagnostic evaluation in primary care.

## Discussion

Our study investigated parents’ and primary health care clinicians’ knowledge and perceptions regarding pediatric hypertension, a topic that has been largely overlooked in existing literature. We specifically sought a target population at high risk: families with children with hypertension not pharmacologically treated and clinical practices with an above average likelihood of diagnosing hypertension. Both groups demonstrated awareness of the importance of addressing pediatric hypertension but exhibited significant mistrust in clinic BP measurements. Clinicians’ downplaying of elevated BP readings and the asymptomatic nature of hypertension contributed to a lack of prioritization and subsequent follow-up care. Both parents and clinicians expressed a strong preference for nonpharmacologic interventions, reflecting concerns about medication adverse effects and long-term impacts. Parents desired comprehensive diagnostic testing, whereas clinicians preferred referring patients to specialists. Although prior studies have acknowledged the perspectives of caregivers, patients, and medical professionals regarding hypertension management, our research provides a deeper understanding of these dynamics through qualitative analysis, capturing a broader range of insights with contextual details not previously addressed cohesively within a single setting.^9,11,19,20,21^

Parents in our study were knowledgeable about hypertension, rooted in their personal or family experiences, recognizing the potential for future organ damage or the insidious nature of hypertension as a silent killer, which aligns with previous research^11,22^; however, prior studies have overlooked how this may impact pediatric hypertension management. In addition, previous studies on the knowledge and perceptions regarding pediatric hypertension have been limited by small samples,^9^ methodologic constraints,^19,21^ inadequate exploration of both health care clinician and family perspectives,^11,19,20^ and exclusion of patients with diagnosed hypertension,^9,19^ thus impeding a comprehensive understanding of how these knowledge and perceptions may lead to low pediatric hypertension detection. Our study highlights a clear connection between parents’ knowledge of hypertension and their personal and familial experiences, rather than knowledge acquired during discussions with clinicians in the clinic. Additionally, parents expressed a sense of self-assurance regarding their knowledge of hypertension, which sometimes hindered them from asking questions when hypertension was detected in their child, potentially missing opportunities for further education on pediatric hypertension (eFigure in Supplement 1). This hesitancy may lead to a lack of understanding about specific hypertension nuances in childhood and its implications for their own child. Clinicians also may assume that because parents have adult relatives with hypertension, they do not require additional education or counseling.

Both parents and clinicians expressed mistrust in clinic BP measurements, but their reasons differed. Parents’ skepticism was influenced by clinicians downplaying high BP readings, showing a lack of confidence in clinic measurements. Clinicians, on the other hand, doubted the accuracy of these measurements and often attributed high readings to white coat syndrome, consistent with a previous study.^9^ Our study contributes to this understanding by revealing how clinician attitudes reinforce parental beliefs that high BP in children may not be a cause for concern, likely diminishing interest in ongoing care, a dynamic not captured in previous studies.^9,11,20^ Lack of belief in BP measurements is a large barrier that needs to be overcome to effectively implement BP screening.

Previous research has noted parents’ reluctance to consider medication for pediatric hypertension,^11^ which resonates with our study’s findings. One study, which focused on pediatric cardiologist perspectives, advocated for primary care clinicians to take on a more active role in hypertension management, including prescribing medications and ongoing monitoring^23^; however, the perspectives and discomfort regarding medical management among primary care clinicians—the first line in the detection of hypertension and decision-makers in this matter—are often overlooked.^23^

In our study, parent concerns about potential adverse effects and personal experiences with medication significantly shape their reluctance toward antihypertensives. In addition, the perspectives of primary care clinicians also shaped how parents perceived antihypertensive medications, which highlights their pivotal role in treatment decision-making. We found that primary care clinicians were reluctant to prescribe medication for hypertension based on concerns about managing adverse effects, limited experience and training in pediatric hypertension management, and lack of patient or parent acceptance. Moreover, parents’ and patients’ lack of acceptance further exacerbates clinicians’ hesitancy to prescribe medications, resulting in a complex interaction that ultimately fuels inadequate medical management of hypertension. Although prior literature has reported on parent^11^ or clinician^9^ reluctance toward medications, the amplification of concerns between parents and clinicians has not been previously reported and is a novel finding of this study.

In our study, parents’ insistence on regular BP checks during annual physical examinations driven by their family history and personal experiences with hypertension underscored their understanding of the importance of early detection and lifestyle modifications. However, clinicians often overlook these concerns, downplaying high BP readings due to health system limitations and cultural norms.^9^ Moreover, structural factors, such as competing demands on clinicians’ time and energy, measurement issues, and lack of emphasis on hypertension within health care systems, may contribute to the deprioritization of pediatric hypertension.^9^ This discrepancy in prioritization between parents and clinicians highlights a crucial difference in shared values likely contributing to low pediatric hypertension detection rates. Additionally, the absence of integrating BP monitoring and counseling into clinician workflows points to structural barriers hindering pediatric hypertension management prioritization.^9,19,20^

In our study, we observed disparities between parents desiring additional testing and clinicians referring patients rather than conducting diagnostic evaluation, a finding not captured in previous studies.^9,11,20^ The current American Academy of Pediatrics hypertension guidelines recommend history, physical examination, and screening chemistry laboratory tests and discourage testing for secondary causes unless something in the above workup produces positive findings. Echocardiography is recommended if hypertension is detected, which reflects the fact that primary, not secondary, hypertension is responsible for most pediatric hypertension.^24^ This finding highlights the need for more recognition of the high burden of primary hypertension in the pediatric population, clear testing guidelines to reduce undertesting or overtesting, improving testing appropriateness, and enhancing parent satisfaction with the management plan.

Our study highlights the crucial role of patient and parent engagement^25^ to enhance service delivery^26^ and actively incorporate parent perspectives, which can significantly influence routine and ongoing care for hypertension. Clinicians should engage proactively with parents, asking open-ended questions about their understanding of hypertension based on family experiences, which can significantly affect their child’s care. For instance, clinicians could delve deeper into parents’ understanding of hypertension based on their experiences (eg, instead of asking, “Do you have a family history of hypertension?” clinicians can ask, “What do you know about hypertension based on your family experiences? How may it impact your child based on your family experiences?”). This shift from closed to open-ended questions encourages parents to share valuable dialogue to address misconceptions. Our study reveals that health care clinicians should recognize how their behavior may inadvertently reinforce parent behavior, including a lack of urgency to act on high BP or attributing high BP to situational factors. Health care clinicians can foster a more collaborative and practical approach to pediatric hypertension care by acknowledging and understanding parents’ concerns and perspectives, including misconceptions (eg, about antihypertensives) and addressing parents’ desire for further diagnostic testing by discussing risks and benefits.

Educational programs targeted to both parents and clinicians can enhance communication strategies, build trust, and improve skills in lifestyle counseling and hypertension management. Updating pediatric hypertension guidelines to incorporate parent and care clinician perspectives, especially regarding disparities in testing prioritization and medication use while promoting lifestyle intervention, is crucial. Guidelines should consider empowering parents to measure their child’s BP at home, providing them with a heightened awareness of their child’s BP readings and better understanding of the validity and significance of the numbers. This involvement can increase parent engagement and create a proactive approach to managing pediatric hypertension. Incorporating an ambulatory BP monitoring system may provide continuous and accurate measurements over time, offering valuable insights into a child’s true BP status.^6,19,20,27^

### Limitations

This study has some limitations. As with qualitative research, the data consisted of self-reported interview responses from parents and health care clinicians, which have a possibility of recall bias or social desirability bias. The study did not include long-term follow-up data, which could provide valuable insights into the dynamic nature of perspectives over time. Additionally, by focusing only on parents who did not follow up after a hypertension diagnosis, we are unable to determine how their beliefs and attitudes differ from those who adhered to follow-up care.

## Conclusions

Our study emphasizes the importance of understanding the perspectives of both parents and health care clinicians on pediatric hypertension to improve detection and management. Enhanced collaboration, communication, and tailored strategies within health care systems can bridge knowledge gaps and optimize pediatric hypertension care. By addressing the concerns and preferences of both parents and clinicians, health care systems can improve hypertension detection rates and patient outcomes, ultimately reducing the long-term health risks associated with pediatric hypertension.

## Notes

Supplement 1. **eTable 1.** Characteristics of Clinical Sites of Health Care Provider Participants **eTable 2.** Consolidated Framework for Implementation Research Used to Develop Parent and Provider Interview Questions **eTable 3.** Major Themes and Their Representative Consolidated Framework for Implementation Research Domains and Constructs **eFigure.** Influences on Parent Perceptions and Behaviors Leading to Low Detection and Management of Pediatric Hypertension

Supplement 2.
Data Sharing Statement
