# Supplementary material for: Parent and Primary Care Clinician Perceptions About Pediatric Hypertension
Source: JAMA Netw Open. 2024 Dec 13;7(12):e2451103. doi: 10.1001/jamanetworkopen.2024.51103 (PMC11645643; doi:10.1001/jamanetworkopen.2024.51103)
Supplement: Supplement 1. — eTable 1. Characteristics of Clinical Sites of Health Care Provider Participants eTable 2. Consolidated Framework for Implementation Research Used to Develop Parent and Provider Interview Questions eTable 3. Major Themes and Their Representative Consolidated Framework for Implementation Research Domains and Constructs eFigure. Influences on Parent Perceptions and Behaviors Leading to Low Detection and Management of Pediatric Hypertension [file jamanetwopen-e2451103-s001.pdf]

Supplementary Online Content

Zaidi AH, Sood E, De Ferranti S, et al. Parent and primary care clinician perceptions about pediatric hypertension. *JAMA Netw Open*. 2024;7(12):e2451103. doi:10.1001/jamanetworkopen.2024.51103

eTable 1. Characteristics of Clinical Sites of Health Care Provider Participants
eTable 2. Consolidated Framework for Implementation Research Used to Develop Parent and Provider Interview Questions
eTable 3. Major Themes and Their Representative Consolidated Framework for Implementation Research Domains and Constructs
eFigure. Influences on Parent Perceptions and Behaviors Leading to Low Detection and Management of Pediatric Hypertension

This supplementary material has been provided by the authors to give readers additional information about their work.

© 2024 Zaidi AH et al. *JAMA Network Open.*

eTable 1. Characteristics of Clinical Sites of Health Care Provider Participants

Site	Number of providers	Child Opportunity Index	Gap in care >1 year	Roles
1	4	Very High	4.2%	2 MD, 1 Nurse Manager, 1 Nurse
2	2	Very High	0.9%	1 MD, 1 Advanced Practice Provider
3	3	Low	7.4%	1 MD, 1 Nurse Manager, 1 Medical Assistant
4	2	Low	5.6%	1 Physician, 1 Nurse Manager
5	2	Moderate/low	8.8%	1 Physician, 1 Nurse
6	2	Moderate/low	3.7%	1 MD, 1 MA
7	3	Low	5.6%	1 MD, 1 Nurse Manager, 1 Medical Assistant
8	2	Low	3.9%	1 MD, 1 Nurse Manager
9	2	Low	28%	1 MD, 1 Nurse
10	3	Low	5.1%	1 MD, 1 Nurse Manager, 1 Nurse

The table represents the recruitment log of healthcare providers based on Child Opportunity Index (COI), the percentage of patients with a hypertension diagnosis with a gap in care greater than 1 year without any follow-up, and healthcare professional role based on site.

© 2024 Zaidi AH et al. *JAMA Network Open.*

eTable 2. Consolidated Framework for Implementation Research Used to Develop Parent and Provider Interview Questions

**CFIR DOMAINS**					
	**Outer Settings**	**Inner Settings/ Organization**	**Innovation**	**Characteristics of the Individual**	**Implementation Process**
**Factors influencing Implementation**	Patient Needs & Resources	Access to Knowledge & Information	Evidence Strength & Quality; Adaptability	Knowledge & Beliefs about Intervention; Self-Efficacy	External Change Agents
**Example(s) of factors**	Patient resources to attend follow-up visits for accurate HTN diagnosis	Ease of access to digestible information and knowledge	The degree to which essential elements of the intervention can be tailored to meet local needs	Provider attitudes towards HTN screening guidelines	Individuals who are affiliated with an outside entity that formally influences or facilitates intervention
**Qualitative Interview Prompt Example**	*Parent:* What makes it difficult for you to follow up?<br><br>*Provider:* What barriers and challenges have you faced when discussing high BP or HTN with the patient and families?	*Parent:* How did you feel about the information you received regarding the BP or the HTN diagnosis from the primary care provider?<br><br>*Provider:* Are there any knowledge gaps for you and your medical assistants and nurses? (If so, what would help to decrease such gaps?)	*Parent:* If you had to improve the detection of HTN, what would you do differently for other families?<br><br>*Provider:* Think about all your competing priorities in the clinic; how important is diagnosis and treatment of HTN compared to other issues?	*Parent:* How comfortable do you feel about criteria for referral after you diagnose HTN?<br><br>*Provider:* Do you think high BP is a problem for children? (If yes, explain why? And if not, why not?)	*Parent:* Would getting your child's BP checked somewhere else, e.g., in school or at home, help?<br><br>*Provider:* How could the sub-specialists help you detect high BP or HTN?

CFIR, Consolidated Framework for Implementation Research; HTN, hypertension; BP, blood pressure

© 2024 Zaidi AH et al. *JAMA Network Open.*

eTable 3. Major Themes and Their Representative Consolidated Framework for Implementation Research Domains and Constructs

MAJOR THEME	CFIR DOMAIN	CFIR CONSTRUCTS
**Knowledgeable about HTN**	Innovation	*Innovation Source:* Both parents and providers demonstrated awareness, indicating credibility in the information about pediatric HTN.<br><br>*Evidence-Base:* Recognition of the significance of pediatric HTN suggests an acknowledgment of robust evidence supporting the importance of addressing this condition.<br><br>*Relative Advantage:* The perceived importance of addressing pediatric HTN by parents implies its superiority over neglecting the condition.<br><br>*Innovation Complexity:* Parents and providers point out the complexity of pediatric HTN, including its asymptomatic nature, reflecting the challenge of addressing this condition.
**Lack of trust in high BP noted in clinic**	Outer setting	*Local Attitudes:* Skepticism towards high BP readings indicates a lack of trust or belief in the significance of these measurements prevalent among parents and providers.<br><br>*Local Conditions:* The contextual factors within clinics influence the perception of a lack of concern with high BP readings for both parents and providers, potentially leading to low pediatric HTN detection.
**Prioritization of pediatric HTN**	Inner setting	*Structural Characteristics:* Provider/clinic prioritization variations may stem from workflow differences or structural factors influencing clinical decision-making.<br><br>*Culture:* Differences in prioritization reflect variations in shared values and norms within healthcare settings.
**Medication use in pediatric HTN**	Characteristics of the individual	*Roles of the Individuals:* Providers' reservations about medication use highlight the influence of their roles and responsibilities in decision-making regarding pediatric HTN management.<br><br>*Characteristics:* Providers' concerns about medication side effects and patient acceptance

© 2024 Zaidi AH et al. *JAMA Network Open.*

		reflect on their capability and motivation to prescribe medications.
**Further testing in pediatric HTN**	Implementation process	*Assessing Needs:* Parents' advocacy for additional tests underscores their perception of the need for comprehensive diagnostic approaches in pediatric HTN management.<br><br>*Tailoring Strategies:* Providers' preference for selective testing reflects adapting diagnostic strategies to fit individual patient contexts.

CFIR, Consolidated Framework for Implementation Research; HTN, hypertension; BP, blood pressure.

© 2024 Zaidi AH et al. *JAMA Network Open.*

eFigure. Influences on Parent Perceptions and Behaviors Leading to Low Detection and Management of Pediatric Hypertension

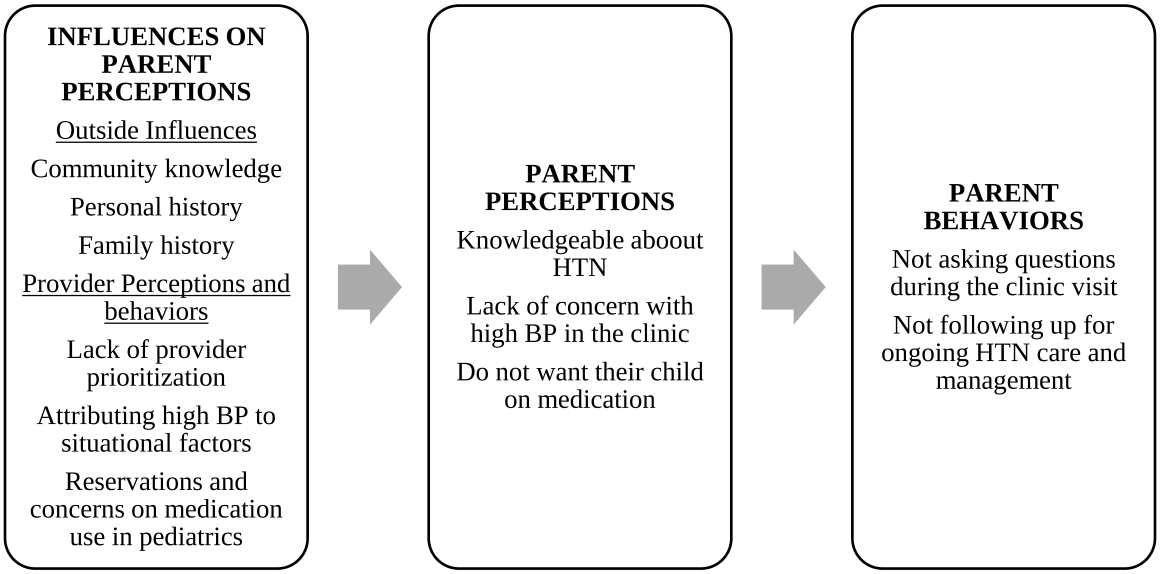

HTN, hypertension; BP, blood pressure.

© 2024 Zaidi AH et al. *JAMA Network Open.*
