# Supplementary material for: Parent and Primary Care Clinician Perceptions About Pediatric Hypertension
Source: JAMA Netw Open. 2024 Dec 13;7(12):e2451103. doi: 10.1001/jamanetworkopen.2024.51103 (PMC11645643; doi:10.1001/jamanetworkopen.2024.51103)
Supplement: Supplement 2. — Data Sharing Statement [file jamanetwopen-e2451103-s002.pdf]

Data Sharing Statement

Zaidi. Parent and Primary Care Clinician Perceptions About Pediatric Hypertension. *JAMA Netw Open*. Published December 13, 2024. doi:10.1001/jamanetworkopen.2024.51103

Data
Data available: Yes
Data types: Deidentified participant data
How to access data: Data requests can be emailed to research coordinator at varsha.zadokar@nemours.org
When available: With publication

Supporting Documents
Document types: Informed consent form
How to access documents: Data requests can be emailed to research coordinator at varsha.zadokar@nemours.org
When available: With publication

Additional Information
Who can access the data: Anyone - Data requests can be emailed to research coordinator at varsha.zadokar@nemours.org
Types of analyses: any purpose
Mechanisms of data availability: signed data agreement
